# Novel Virostatic Agents against Bluetongue Virus

**DOI:** 10.1371/journal.pone.0043341

**Published:** 2012-08-15

**Authors:** Linlin Gu, Volodymyr Musiienko, Zhijun Bai, Aijian Qin, Stewart W. Schneller, Qianjun Li

**Affiliations:** 1 Jiangsu Key Laboratory of Preventive Veterinary Medicine, Yangzhou University, Yangzhou, China; 2 Division of Infectious Diseases, Department of Medicine, University of Alabama at Birmingham, Alabama, United States of America; 3 Molette Laboratory for Drug Discovery, Department of Chemistry and Biochemistry, Auburn University, Auburn, Alabama, United States of America; 4 Guangzhou Center for Disease Control and Prevention, Guangdong, China; University of Pittsburgh, United States of America

## Abstract

Bluetongue virus (BTV), a member in the family *Reoviridae*, is a re-emerging animal disease infecting cattle and sheep. With its recent outbreaks in Europe, there is a pressing need for efficacious antivirals. We presented here the identification and characterization of a novel virostatic molecule against BTV, an aminothiophenecarboxylic acid derivative named compound 003 (C003). The virostatic efficacy of C003 could be improved *via* chemical modification, leading to a *de novo* synthesized compound 052 (C052). The 50% effective concentrations (EC_50_) of C003 and C052 were determined at 1.76±0.73 µM and 0.27±0.12 µM, respectively. The 50% cytotoxicity concentration (CC_50_) of C003 was over 100 µM and the CC_50_ of C052 was at 82.69 µM. Accordingly, the 50% selective index (SI_50_) of C003 and C052 against BTV was over 57 and 306, respectively. The inhibitory effect of C003/C052 on BTV-induced apoptosis was also confirmed *via* the inhibition of caspase-3/-7 activation post BTV infection. C003/C052 could inhibit BTV induced CPE even when added as late as 24 h.p.i., indicating that they might act at late stage of viral life-cycle. C003/C052 could reduce over two-logs of both the progeny virus production and the number of genomic viral RNA copies. Interestingly, both the activation of host autophagy and viral protein expression were inhibited post BTV infection when cells were treated with C003 and C052, suggesting that C003/C052 might act as virostatic agents *via* inhibiting host autophagy activation. Although further investigations might be needed to pin down the exact mechanism of C003/C052, our finding suggested that these compounds might be potent lead compounds with potential novel mechanism of action against BTV.

## Introduction

Bluetongue virus (BTV), a double-strand RNA (dsRNA) virus, is the prototype virus in the genus *Orbivirus* within the family *Reoviridae*. The segmented BTV dsRNA genome encodes ten viral proteins (VP), including seven structural proteins (VP1-7) and three non-structural (NS) proteins, NS1, NS2 and NS3 [1], [2], [3]. As a member of arboviruses, BTV is transmitted by certain species of *Culicoides* biting midges, including *C. variipennis* and *C. imocola* [4], [5]. Bluetongue disease is a non-contagious viral disease affecting domestic animals including sheep and cattle primarily, as well as wild ruminants such as buffalo, antelope, deer, elk and camels. BTV disease is one of the most important diseases of domestic livestock, causing $3 billion per year loss worldwide [6], [7]. In sheep, the disease is acute, and mortality is accordingly high [8]. BTV is listed under the Office International des Epizooties (OIE) Terrestrial Animal Health Code –2009 [2]. Exotic BTV is also listed in the “USDA High Consequence Livestock Pathogens." Due to its economic significance, BTV has been the subject of extensive molecular virology and structural biology studies [9], [10], [11]. The recent development of the BTV reverse genetics system facilitates our understanding toward the BTV viral life-cycle, the structural and functional interrelationship among viral proteins [12], [13], [14]. Hence, BTV is now one of the well-characterized viruses [6], [9], [15].

The main prevention and control measures in endemic areas include active surveillance programs, animal quarantine and movement restrictions, vaccination and insect control measures [2], [16], [17], [18]. Vaccination is used as the most effective and practical measure to minimize losses related to BTV disease and to potentially interrupt the cycle from infected animal to vector [18], [19], [20]. However, due to the complexity of the virus, including the twenty-four different serotypes that have the ability of causing variable diseases, BTV is still endemic in many regions despite the high vaccine coverage in sheep and cattle [21], [22], [23], [24]. The 1998–2001 outbreak of BTV-8 in the Mediterranean Basin is the greatest epizootic of the disease on record, showing that BTV has extended its range northwards into areas of Europe that were never affected before, and has since persisted in many of these locations [6], [25], [26], [27]. In 2006, BTV-8 has reached three northern Europe countries, including the Netherlands, Belgium, and Germany [28], [29], and further spread to surrounding countries, reaching Switzerland by the end of October 2007 [6], [30], [31], [32].

Vaccination of individuals/animals during an outbreak can prove effective, however, protection of an individual/animal from the threat may not occur for two or more weeks after the initial vaccination [33]. Hence, only a drug can be offered as a therapeutic treatment of individuals/animals in an endemic area [33]. Surprisingly, there are no antiviral currently available against BTV disease. Recently, anti-BTV drug discovery has been implemented and potential antiviral lead compound(s) has been identified [34]. The development and validation of a cytopathic effect (CPE)-based assay led to the screening of the NIH Molecular Libraries Small Molecule Repository (MLSMR), with 194,950 small molecule compounds then. Further studies, using various primary, secondary and confirmatory assays, confirmed 185 structures that were grouped into six analog series corresponding to six scaffolds enriched within the active set compared to their distribution in the library [34]. Based on the results from the previous studies [34], we selected and evaluated the virostatic efficacy of a cluster of active compounds, in particular, the aminothiophenecarboxylic acid derivatives. Furthermore, aiming to understand their mechanism of action, various studies were carried out to determine which viral life stage(s) these compounds acted on, focusing on how these virostatic compounds protect cells from BTV-induced apoptosis.

## Results

### Thiophene Derivatives as Lead Virostatic Agents against BTV

**Figure 1:**
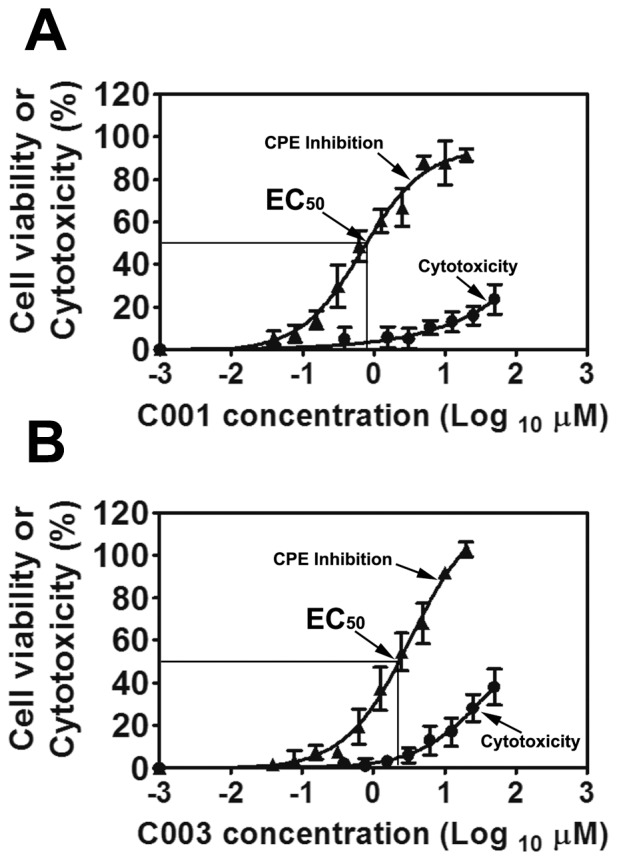
The virostatic efficacy and cytotoxicity were showed in (A) C003 and (B) C001. Cells were infected with BTV at MOI of 0.01 with or without C003 or C001 at various concentrations. At 72 h.p.i, cell viability (triangles) or cytotoxicity (circles) was measured using the CellTiter-Glo reagent. Each data point represents means and standard deviation (SD) from five replicates.

Based on the previously identified six compound clusters [34], we selected 2–4 representative compounds from each cluster and assessed their virostatic efficacies against BTV, including the 50% effective concentration (EC_50_) and the 90% effective concentration (EC_90_). The EC_50_ of these lead compounds were determined between 0.7 µM and 20 µM, respectively (results not shown). Interestingly, the four thiophene derivative compounds from cluster I, i.e. compound 001 through 004 (C001, C002, C003 and C004), showed potent virostatic efficacy against BTV, especially the aminothiophenecarboxylic acid derivatives C001 and C003. Using the ten-concentration dose response assay, the EC_50_s of C001 and C003 were determined at 0.69±0.15 µM and 1.76±0.73 µM, respectively (Fig. 1A and B). The EC_90_s of C001 and C003 were at 6.20±1.39 µM and 15.86±6.56 µM, respectively (Fig. 1A and B). The above results indicated that both C001 and C003 could be potential novel virostatic agents against BTV. Additionally, the 50% cytotoxicity concentration (CC_50_) of both C001 and C003 were over 100 µM (Fig. 1A and B). Accordingly, the selective indexes (SI_50_), which equals to EC_50_/CC_50_, were above 145 and 57 for C001 and C003, respectively, indicating that both compounds were highly selective against BTV. Considering the compound solubility and availability, we selected C003 as the lead virostatic agent for further evaluation, including structure-activity relationship (SAR) analysis, chemical modification and mechanism of action studies.

### Improving C003 Virostatic Efficacy *via* Chemical Modification

**Figure 2:**
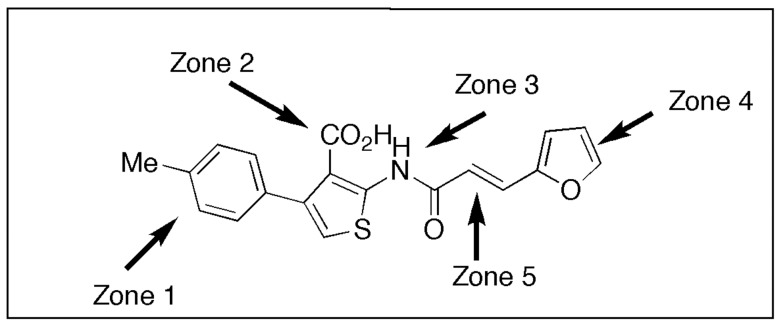
The compound structure of C003 was showed. The compound was divided into five zones as indicated.

**Figure 3:**
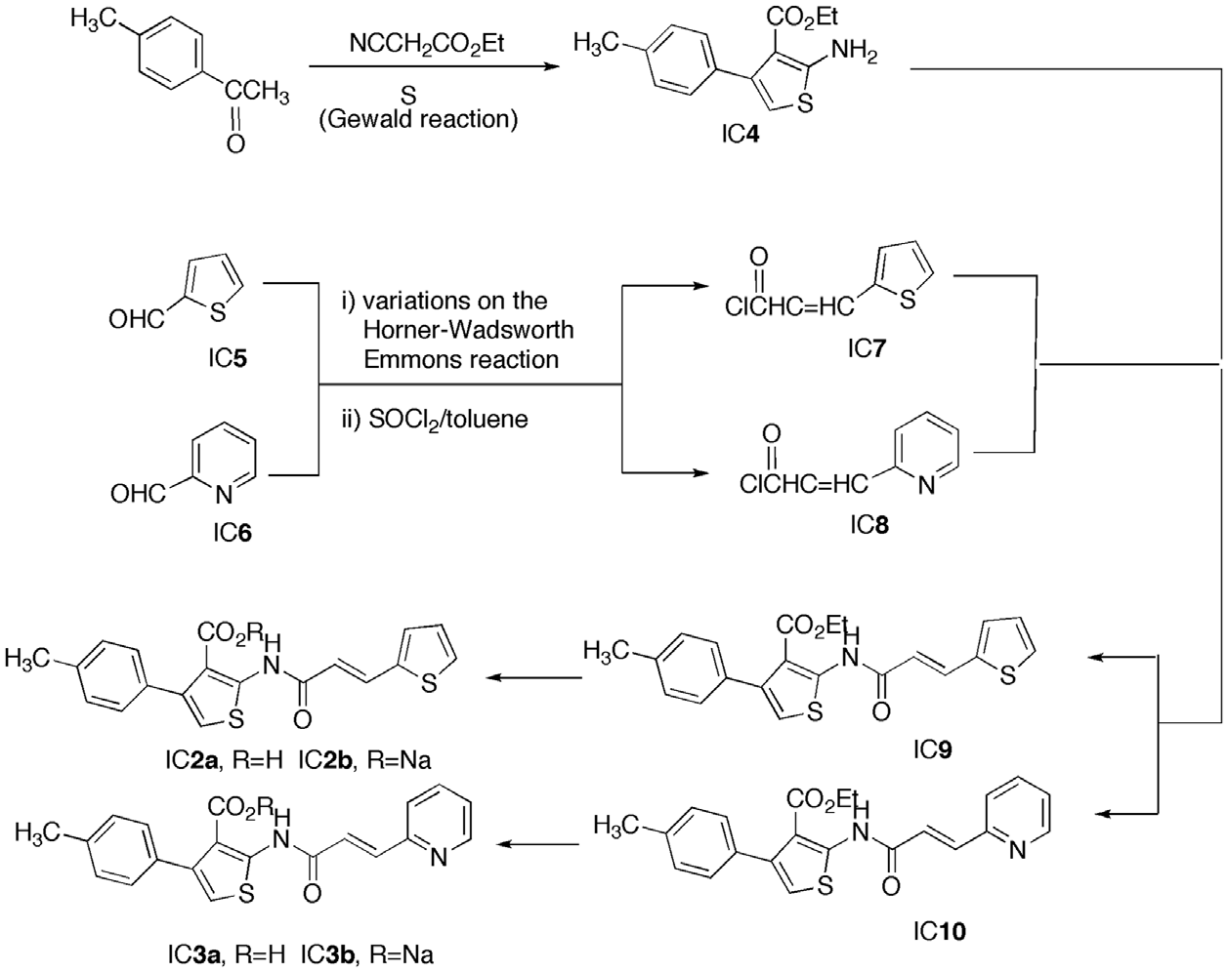
The *de novo* synthesis of C052 (2a) and C055 (3a) were illustrated. Each number here represents each intermediate compound (ic) as described in the text.

C003, or 2-{[3-(2-furyl)acryloyl]amino}-4-(4-methylphenyl)-3-thiophenecarboxylic acid, is an aminothiophenecarboxylic acid derivative with a structure showed in Figure 2. To improve the potential of C003 as an anti-BTV lead structural framework, it was divided into 5 zones for synthetic target goals. Figure 3 illustrates our results from a variation in zone 5 by replacing the furan of C003 with a thiophene (IC**2a or** C052) and pyridine (IC**3a or** C055).

Our approach to C052 (IC**2a)** and C055 (IC**3a)** involved a convergent pathway whereby (1) the Gewald reaction was employed for the thiophene unit (IC**4** of zone 1) and (2) a Horner-Wadsworth-Emmons reaction of 2-thiophenecarboxaldehyde (IC**5**) and 2-pyridinecarboxaldehyde (IC**6**) with malonic acid followed by acid chloride formation provided a means to IC**7** and IC**8**, the zone 5 modifiers. Bringing these units (IC**4** and IC**7/**IC**8**) together produced IC**9** and IC **10**. Saponification of IC**9** and IC**10** gave the sodium salts IC**2b** and IC**3b**, which, upon acidification, yielded the desired final products C052 (IC**2a)** and C055 **(**IC**3a)** (Fig. 3).

**Figure 4:**
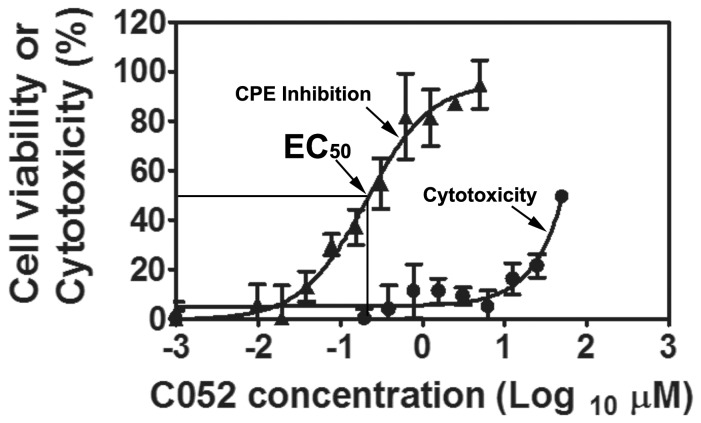
The virostatic efficacy and cytotoxicity of C052 were showed as cells were infected with BTV at MOI of 0.01 in the presence of various concentrations of C052. At 72 h.p.i, cell viability (triangles) or cytotoxicity (circles) was determined using CellTiter-Glo reagent. Each data point represents means and SD from five replicates.

Because of their potential as prodrugs of C052 (IC**2a)** and C055 (IC**3a)**, esters IC**9** and IC**10**, were also evaluated for their virostatic efficacies. Interestingly, while other analogs including C055 did not show any improvement of its virostatic efficacy, the *de novo* synthesized analogs, C052, showed an improved virostatic efficacy with EC_50_ at 0.27±0.12 µM and EC_90_ of 2.5±1.04 µM, respectively (Fig. 4). Similarly, C052 showed very little toxicity with CC_50_ at 82.69 µM. Consequently, the SI_50_ of C052, were at 306. Comparing with the EC_50_ and SI_50_ of C003, the EC_50_ of C052 were 6.5 times lower, and the SI_50_ was 5.4 times higher, indicating that the virostatic efficacy of C003 could be further improved *via* SAR and chemical modifications, leading to a more potent and selective virostatic agent against BTV.

### Both C003 and C052 Prevented BTV Induced CPE

**Figure 5:**
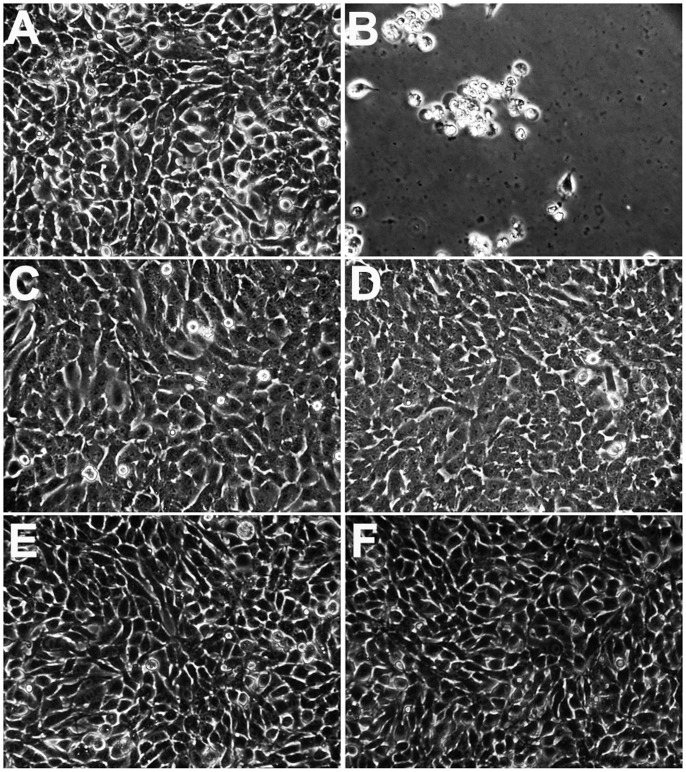
Both C003 and C052 protects BSR cells from BTV induced CPE/apoptosis. In mock infected BSR cells (A), cells formed a monolayer with no cells detached from the plates. At 72 h.p.i., BTV infected cells showed CPE/apoptosis and were detached from the plate (B). When C003 at 10 µM was added to the BSR cells without BTV infection, there were no obvious changes in term of cell morphology (C). There were also no morphology changes when cells were treated with C003 and with BTV infection (D). Similarly, when C052 at 2.5 µM was added to the BSR cells without BTV infection, there were no cytotoxicity (E), and C052 could also block BTV-induced CPE/apoptosis (F).

The CellTiter-Glo cell viability assay determines the number of viable cells in culture based on quantitation of cellular ATP presented, which signals the presence of metabolically active living cells [34], [35], [36]. To further confirm that C003 or C052 could prevent BTV-induced CPE, cell morphology changes at different h.p.i. were examined post BTV infection at MOI of 0.01. Without C003 or C052 treatment, CPE was observed as early as 48 h.p.i. in infected cells. At 72 h.p.i., the majority of infected cells were dislodged from the plate indicating CPE and cell lysis (Fig. 5B). When C003 at 10 µM or C052 at 2.5 µM was added to BTV-infected cells at 0 h.p.i., CPE was not observed up till 72 h.p.i. There was no sign of cell dislodging and lysis (Fig. 5D and F). Similar to previous observation, uninfected cells treated with C003 at 10 µM or C052 at 2.5 µM (Fig. 5C and E) showed no overt toxicity, with regard to cell division, morphology changes, with comparable cell viability to that of the control cells (Fig. 5A). This result confirmed that BTV-induced CPE was prevented after cells were treated with C003 or C052.

### C003 and C052 Prevented BTV-induced Apoptosis

**Figure 6:**
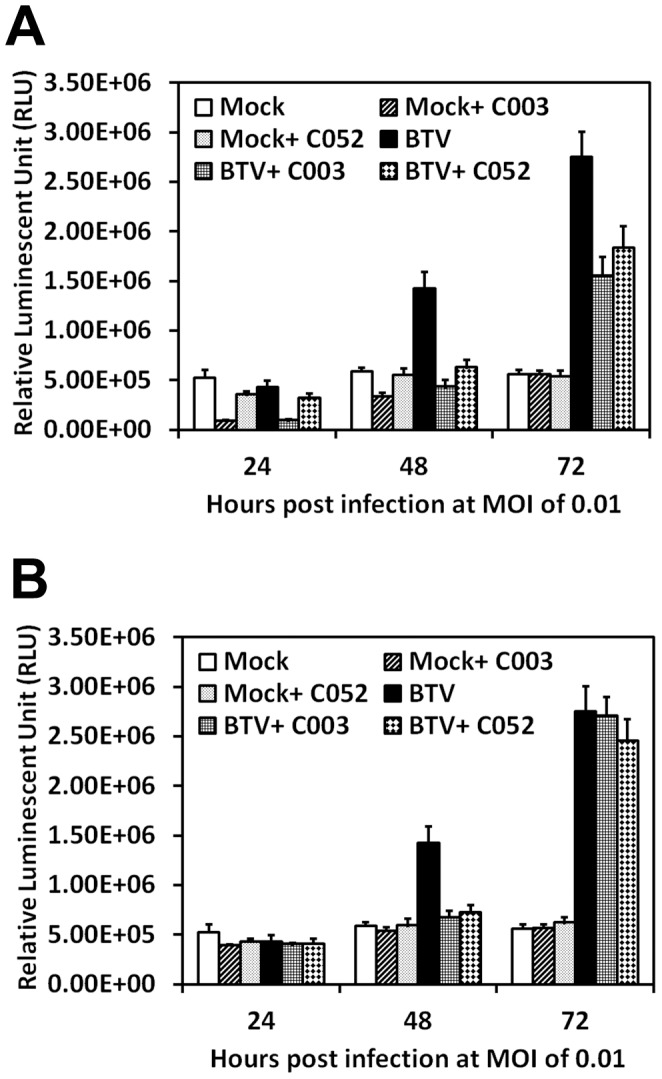
Effects of C003 and C052 on BTV-induced caspase-3/7 activation. BSR cells were mock infected or infected with BTV at MOI of 0.01, and treated with C003 or C052 at their EC_90_ concentrations (A) or EC_50_ concentrations (B). At 24, 48 and 72 h.p.i, caspase-3/-7 activities were determined using the Caspase-Glo-3/7 reagent, and presented as relative fluorescent unit (RLU). Each data points represented mean values and SD from triplicates experiments.

Previous studies have showed that BTV infection in mammalian cells triggers apoptosis leading to CPE/cell lysis *in vitro* and the virus-induced pathogenesis *in vivo* [1], [37]. In order to further confirmed that C003 and/or C052 prevented BTV-induced CPE *via* blocking apoptosis, we examined the activation of caspase-3/7 in infected cells treated with the virostatic compounds at different h.p.i. Our result showed that BTV-induced caspase-3/7 activation, the main characteristics of apoptosis, was inhibited in C003/C052 treated cells. When BTV-infected cells were treated with C003 at 1.8 µM and 10 µM, there were 2.1- and 3.3-fold reductions of caspase-3/7 activity at 48 h.p.i, respectively, comparing with the caspase-3/7 activity in cells infected with BTV but without C003 treatment (Fig. 6A and B). When cells were treated with C052 at 0.27 µM and 2.5 µM, the inhibitions of caspase-3/7 activity were similar to the inhibition of C003 at similar concentrations (1.8 µM and 10 µM), including 2.0- and 2.3-fold decreases of caspase-3/7 activity at 48 h.p.i. (Fig. 6A and B). The above results indicated that C003 and/or C052 could inhibit BTV-induced caspase-3/7 activation, which further prevented BTV-induced apoptosis, consequently CPE and cell lysis.

### The Effect of C003 and C052 on Virus Progeny Production

**Figure 7:**
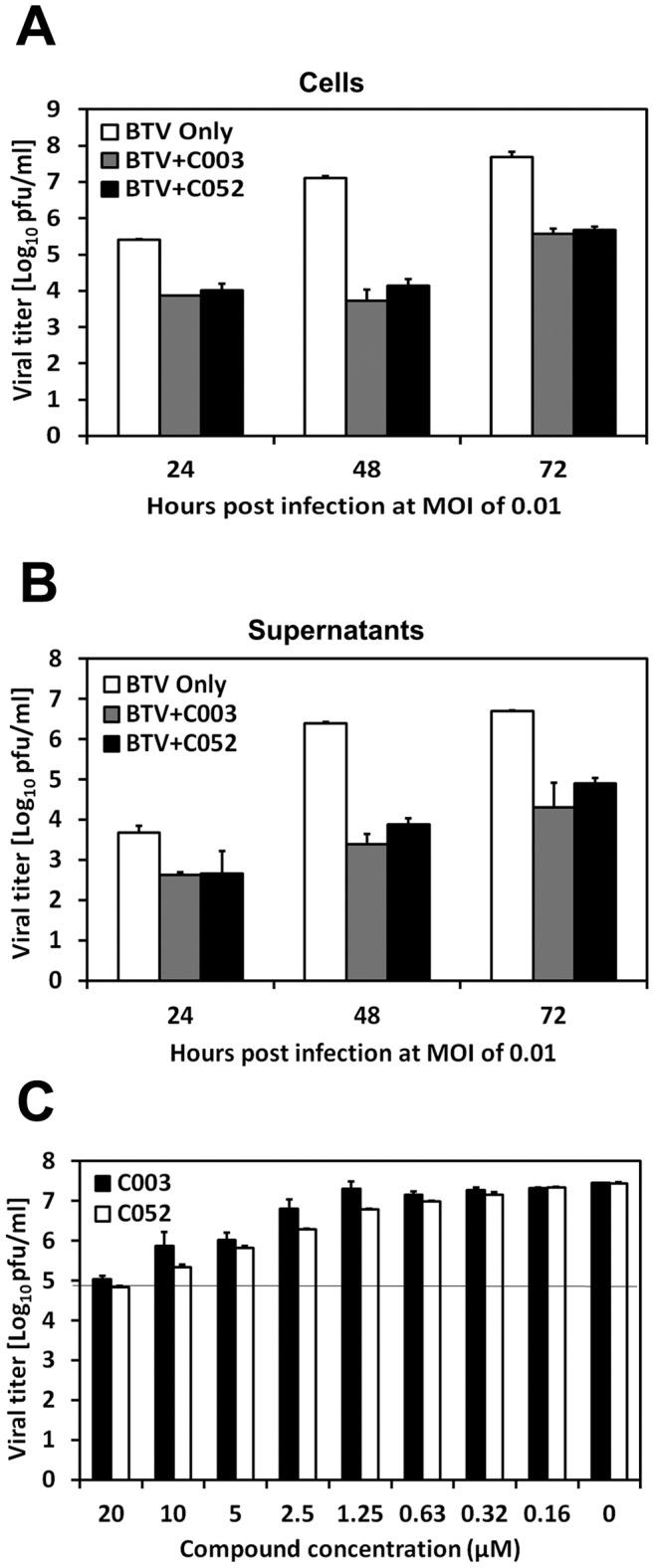
The effects of C003 and C052 on BTV progeny production. BTV infected cells were treated with C003 (10 µM) or C052 (2.5 µM). At different time post infection as indicated in figures, cell (A) and supernatant (B) samples were collected separately. BTV progeny productions were evaluated using the standard plaque assay. In a separate experiment, the plaque reduction assay (C) was carried by mixing C003 or C052 with the overlay and applied directly to BTV-infected cells. At 72 h.p.i, plaques were counted and analyzed. Each data points represented the average values and SD from of three independent replicates.

To understand whether C003 and C052 could disrupt the productive BTV viral life-cycle, we analyzed their effect on BTV progeny productions. BSR were infected with BTV at MOI of 0.01 and cells were treated with C003 or C052 at 10 µM or 2.5 µM, respectively. At different h.p.i, cells and supernatants were collected, and BTV titers in these samples were determined by the conventional plaque assay. At 24 h.p.i., there were one log differences of virus progeny production in cells treated with C003 or C052 at 10.0 µM and 2.5 µM, respectively, when compared with that in samples collected from BTV-infected only cells. There were three- or two-log reductions of virus progeny production in samples collected at 48 h.p.i. and 72 h.p.i. after treated with C003 or C052, respectively (Fig. 7A). Similarly, the reduction of progeny virus production was also noticeable in the supernatant samples after treated with C003 or C052, with three- or two-log reductions of virus progeny titer, respectively, comparing with that in samples collected from BTV-infected supernatant without virostatic compound treatment (Fig. 7B).

The inhibition of C003 and C052 to BTV virus progeny production was further confirmed using the plaque reduction assay. C003 or C052 at different concentration were mixed with the agrose overlay and applied to BTV infected cells (MOI of 0.01) directly. The inhibition of BTV plaque formation was then examined at 72 h.p.i. The effects of C003 and C052 on the BTV plaque reduction were in a dose-dependent fashion. At 72 h.p.i., a one-log reduction of plaque formation was observed, when C003 at 2.5 µM and C052 at 1.25 µM were applied to the BTV infected cells, respectively. When applied at higher concentrations, i.e. 20.0 µM of C003 or C052, to the infected cells, more than two-log of plaque reductions were observed (Fig. 7C). There was no difference in terms of the size or formation of the plaques (results not shown). This result showed that C003 and C052 could reduce the viral plaque formation directly.

### Time-of-addition Assay

**Figure 8:**
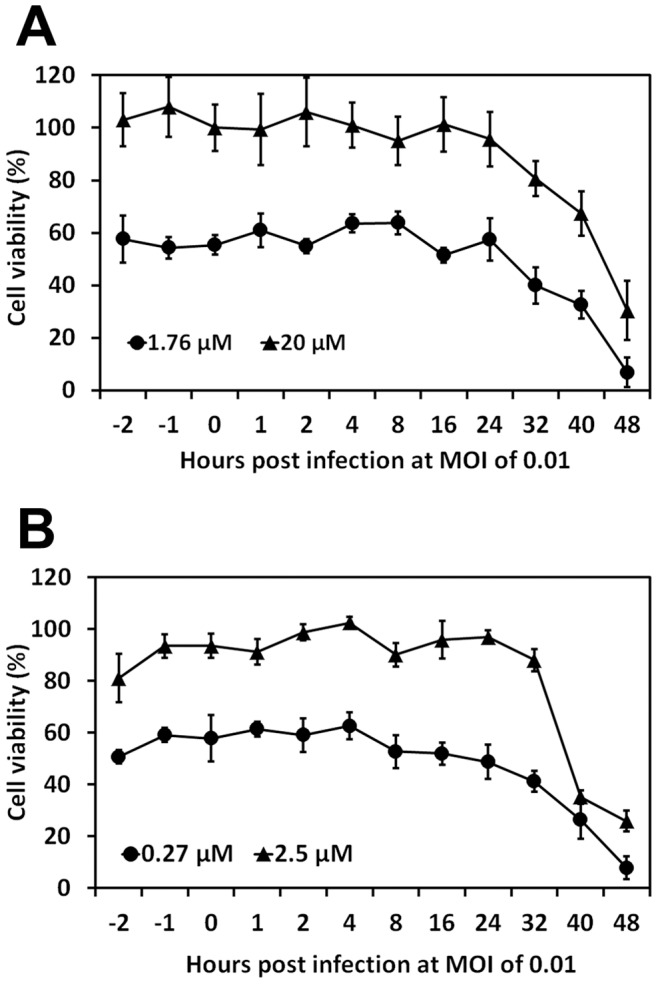
The time-of-addition assay for C003 (A) and C052 (B). C003 at 20 and 1.76 µM, and C052 at 2.5 µM and 0.27 µM, respectively, were added to BTV infected cells at different h.p.i. as indicated, and the protection of C003 and C052 against BTV induced CPE, or cell viability, was measured using CellTiter-Glo reagent at 72 h.p.i. Each data points represented the average values and SD from of eight independent replicates.

Since C003/C052 showed no overt toxicity *in vitro*, yet prevented BTV induced CPE/apoptosis/cell lysis and reduced the virus progeny production, we initiated the mechanism of action studies with the time-of-addition assay to determine the possible stage(s) of viral life-cycle that could be affected by C003 and C052. In order to observed both the enhancement and reduction of virostatic efficacy at different time of addition, we used two concentrations of virostatic compound for both C003 and C052, i.e., the C003 at 1.76 µM and 20 µM, and C052 at 0.27 µM and 2.5 µM, respectively. We first observed that adding virostatic compound C003 or C052 at 1 or 2 hours prior to BTV infection, i.e. −1 and −2 h.p.i., did not change their virostatic efficacies. This result indicated that both C003 and C052 might not act on the early stage of viral life-cycle, such as virus entry (Fig. 8A and B). Interestingly, when the compounds were added to BTV infected cells as later as 24 h.p.i., both C003 and C052 showed similar protection in term of the percentage of viable cells. When added at 32 h.p.i., the percentage of viable cells decreased in both C003 and C052 treatment samples, comparing with their protection in the initial 24 h.p.i., indicating that both C003 and C052 were less protective post certain stage(s) of viral life-cycle. When C003 and C052 were added at 48 h.p.i., both compounds showed no protection to BSR cells from BTV-induced CPE (Fig. 8A and B). Since the first cycle of BTV viral replication usually completed in infected cells within 24 h.p.i., our results suggested that C003 and C052 might not act at the early stage of BTV viral life-cycle, including virus entry and uncoating, but rather at the late stage of BTV viral life-cycle, such as virus replication, packaging, maturation and egress. Meanwhile, it is also possible that these compounds may act on host cellular machineries that were involved during late viral life-cycle.

### Effect of C003 and C052 on BTV Viral Genomic RNA Replication

**Figure 9:**
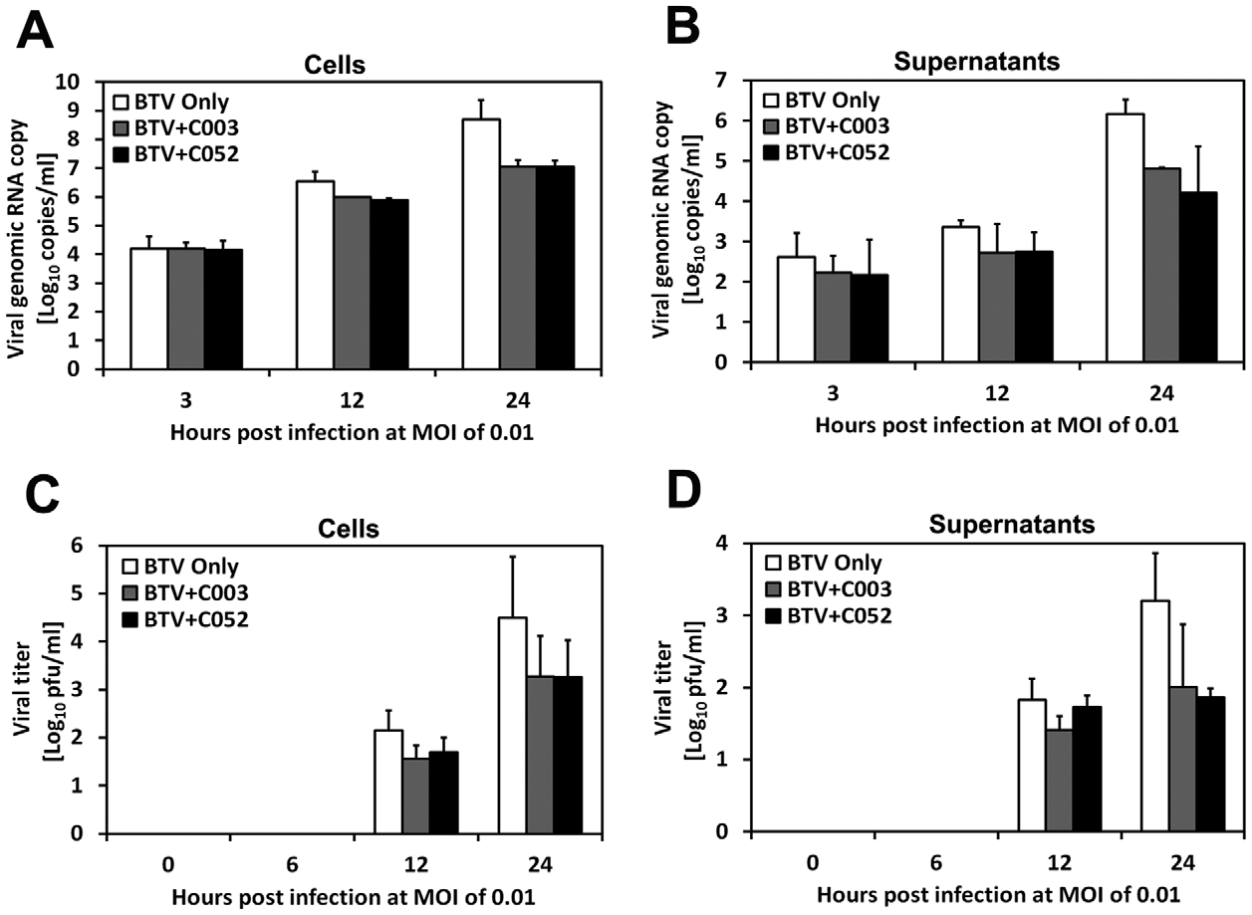
The effects of C003 and C052 on BTV viral genomic RNA synthesized and progeny virus production at MOI of 0.01. BTV infected cells were treated with C003 (10 µM) or C052 (2.5 µM), at different h.p.i. as indicated in figures. The viral genomic RNA was extracted and the copy number was determined using real-time qRT-PCR in cell (A) and supernatant (B) samples. The progeny virus titer in cells (C) and supernatants (D) were also analyzed using standard plaque assay.

To determine whether C003/C052 acts on BTV viral life-cycle directly, we examined the effect of C003/C052 on viral genomic RNA replication in infected cells. BTV viral genomic RNA copies were determined in both cell and supernatant samples using the real time qRT-PCR (Fig. 9A and B). The correspondence of viral genomic RNA replication and virus progeny production in these samples were confirmed by evaluating the amount of infectious virus using standard plaque assay (Fig. 9C and D). Samples were collected post BTV infection and divided into cells and supernatants portion at different time in the initial 24 h.p.i., with and without treatments of C003 (10 µM) and C052 (2.5 µM), respectively. When infected at low MOI (0.01), the viral genomic RNA copies and progeny virus titers obtained from in cell samples represent the events occurred inside the cells for the initial viral-life cycle, including virus entry, replication and maturation within the cells. The viral genomic RNA copies and virus titers in the supernatant samples denotes the events post virus life-cycle inside the cells in the initial 24 h.p.i., including the amount of viruses exited from the cells. At early time points including 3 and 6 h.p.i., there were no differences in viral genomic RNA copies in samples collected from both supernatant and cells, whether treated with C003/C052 or not, indicating that early viral life cycle, such as the entry of BTV into the cells, were not interfered (Fig. 9 A and B). Meanwhile, there is no detectable progeny virus production in all samples (Fig. 9C and D). At 24 h.p.i., there were pronounced reductions, around two-log, of virus genomic RNA copies and virus titers in supernatant samples with C003/C052 treatment, when compared with these in the supernatant samples without C003/C052 treatment. In supernatant samples collected at 24 h.p.i from BTV infected but without C003/C052 treatment, there were 6.17×10^6^ copies of BTV viral genomic RNA, while there were only 4.81×10^4^ or 4.22×10^4^ copies of BTV viral genomic RNA in samples treated with C003 or C052, respectively (Fig. 9B). This result indicated a pronounced effect of these virostatic agents at the late stage of viral life-cycle. However, C003/C052 might also inhibit virus replication directly, since there was a one-log difference in the viral genomic RNA copies and virus titers when comparing samples from infected cells with and without C003/C052 treatments (Fig. 9A).

### Effect of C003/C052 on VP6 Expression

**Figure 10:**
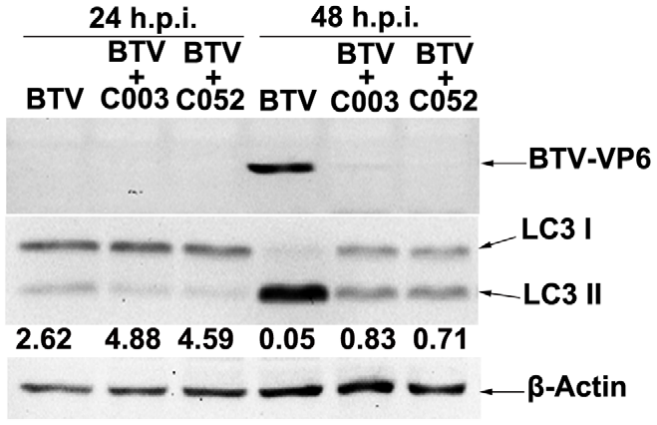
Effects of C003 and C052 on BTV VP6 protein expression and LC3-I/LC3-II conversion were analyzed in sample collected at different h.p.i. Cells were infected with BTV at MOI of 0.01, and treated with C003 (10 µM) or C052 (2.5 µM). VP6 expressions and LC3-I/LC3-II conversion in these samples were determined using the western blot.

To further confirm the effect of C003/C052 on BTV viral life-cycle, we also analyzed viral protein synthesis in the above cell samples infected at MOIs of 0.01, with and without C003 or C052 treatments. VP6 of BTV possesses nucleoside triphosphatase, RNA binding, and helicase activities [9], [11], [38]. For a successful BTV life-cycle, VP6 is an integral part of a transcription complex essential for primary replication [14]. At MOI of 0.01, VP6 was not detectable at 24 h.p.i. in all samples, but was detected at 48 h.p.i. in BTV-infected cells without C003/C052 treatment (Fig. 10). When BTV-infected cells were treated with C003 at10 µM and C052 at 2.5 µM, respectively, there were either very low or no detectable VP6 expression in the cells at 48 h.p.i. (Fig. 10). This result supported our previous observations that C003/C052 reduced viral protein expression when infected at low MOI of 0.01.

### Effect of C003 and C052 on BTV-induced Autophagy

Based on the data presented above, we hypothesized that C003/C052 might act on host machineries/pathways involved in the viral life-cycle, including the autophagic pathway. Autophagy is one of the major pathways for the degradation and turnover of long-lived proteins and organelles in cells during cell starvation or upon various stimuli, such as virus infection [39], [40], [41], [42]. Growing evidences suggest that autophagy actively participates in various pathogenic infections, including the formation of autophagosome for the replication of Influenza A virus [43], [44], [45], hepatitis C virus [46], [47] and Dengue virus [48]. The turnover of microtubule-associated protein light chain 3-I (LC3-I) to LC3-II, or the ratio of LC3-I and LC3-II, is the only biomarker that is reliably associated with the formation of autophagosome. This is also related to virus replication and the leading indicator of virus-induced apoptosis. Since C003 and C052 prevented BTV-induced apoptosis, we examined the activation and formation of autophagy in BTV-infected cells, with and without virostatic compound treatment. When infected at MOI of 0.01, autophagy was not activated at 24 h post BTV infection as the level of LC3-II showed no difference in sample with or without C003/C052 treatment. However, at 48 h.p.i., there was a turnover of LC3-I to LC3-II in BTV infected cells without C003/C052 treatment, with the LC3-I/LC3-II ratio at 0.05 which indicating most of LC3-I was cleaved into LC3-II (Fig. 10). The turnover of LC3-I to LC3-II was at a much lower level when C003 at 10µM or C052 at 2.5 µM was added to the BTV-infected cells, with the ratio of LC3-I/LC3-II at 0.83 and 0.71 respectively (Fig. 10). This result suggested that C003/C052 might act on interfering with BTV induced autophagy, consequently, inhibiting BTV-induced CPE.

## Discussion

As a result of its economic significance and as a model system, BTV has been the subject of extensive molecular, genetic and structural studies, and now represents one of the most well characterized viruses [7], [10], [11], [15]. Much of the efforts were focused on developing vaccines against BTV, including the attenuated BTV and VLPs [18], [49], [50]. In contrast, it remains under-investigated to utilize the knowledge from the molecular and virological studies for antiviral drug discovery. Here, we reported the characterization of a novel class of compound as virostatic agents against BTV. These compounds, i.e. the thiophene derivatives, have been identified as active compounds in various assays, including as an enhancer of survival motor neuron protein splice variant expression; an activator for alpha-synuclein 5′UTR - 5′-UTR binding, and for STAT1 activation [51]. It is plausible that the aminothiophenecarboxylic acid derivative–C003 was identified as a novel compound against BTV, with potent virostatic efficacy, low cytotoxicity and high SI. More interestingly, the preliminary SAR analysis of C003 leads to an improved virostatic compound–C052, via *de novo* synthesis. Our studies suggest that using C003/C052 as lead compounds, it is possible to develop novel and potent antivirals against BTV *via* chemical modification, while maintain its low toxicity and high selectivity.

For the majority of RNA viruses, the induction of apoptosis, which facilitates the virus dissemination, is an essential step in the late stage of viral life-cycle. Interfering the host apoptotic machinery, especially inhibiting the virus induced apoptosis, will disrupt the successful viral life-cycle via blocking virus release [52], [53], [54]. Thus, host apoptotic response post virus infection is an attractive target for antiviral drug development because it is a common and indispensible late step for a productive viral life-cycle of many, if not all, viruses. Previous studies have shown that BTV infection triggers apoptosis via both intrinsic and extrinsic pathways [1], [37], [55]. The induction of apoptosis is involved in BTV-induced pathogenesis *in vivo* [56], [57]. Our mechanism of action studies indicated that C003 and C052 were active by protecting cells from BTV-induced apoptosis. The inhibition of caspsse-3/7 activation indicated the targets should be in the upstream of the cascade of caspase activation. However, when BSR cells were treated with pan-caspase inhibitor, including Z-VAD-FMK, cells were not protected from BTV-induced apoptosis. BTV-induced CPE was observed, and BTV replication was not interrupted (results not shown). This suggested that while apoptosis/CPE was inhibit by C003/C052, the mechanism of action of these virostatic compounds might not directly related to BTV-induced apoptotic pathways, rather as a results of inhibiting a late step in viral life-cycle or host machineries inducing upstream apoptotic signals.

Interestingly, our results showed that the host autophagy machinery/pathway was inhibited by C003 and/or C052, as LC3-II, the only reliable autophagy marker, was down-regulated when BTV-infected cells were treated with these compounds (Fig. 10). Autophagy is a highly conserved process in eukaryotes in which the cytoplasm, including excess or aberrant organelles, is sequestered into double-membrane vesicles and delivered to the degradative organelle, the lysosome/vacuole, for breakdown and eventual recycling of the resulting macromolecules [44], [47], [58]. Autophagy is activated as an adaptive response to a variety of extracellular and intracellular stimuli, including nutrient deprivation, hormonal or therapeutic treatment, aggregated and misfolded proteins, damaged organelles and microbial infection [58], [59], [60]. Although the autophagy pathway might be a host defense mechanism, different viruses have developed various strategies to counteract these antiviral mechanisms, and/or to utilize the autophagy machinery as proviral host factors favoring viral replication [46], [61]. Recently, a growing list of viruses has been shown to be targeted for autophagic degradation [62]. For instance, autophagy is actively involved in influenza A virus replication by triggering autophagosome formation, increasing the level of LC3-II and enhanced autophagic flux [45]. Dengue virus can also activate autophagic machinery that is favorable for viral replication [48], [63], and autophagosomes act as a site for translation and replication of dengue virus-2 and that its entry and replication are linked through an ongoing association with membranes of an endosomal–autophagosomal lineage [64], [65]. Similarly, induction of autophagy pathways can enhance the replication of poliovirus and coronavirus [66], [67]. During the infection of dsRNA virus rotavirus, nonstructural protein 4 (NSP4) colocalized with LC3 in cap-like structures associated with viroplasms, the site of nascent viral RNA replication, suggesting a possible new mechanism for the involvement of NSP4 in virus replication [68], [69]. Our finding showed that C003 and C052 interacted with autophagy machinery, which may lead to a new research avenue to develop novel antivirals intervene virus-autophagy interactions.

While virus entry and virus replication have been targets for extensive antiviral drug discovery and development, egress/dissemination of a newly synthesized progeny virus, equally important in relation to successful viral infection, receives much less attentions [70]. This is mainly due to the lack of information on the mechanism of egress/dissemination. Interestingly, further studies showed that autophagy may assist poliovirus egress. During Poliovirus and Coxsackievirus B3 infection, the co-localization of non-structural proteins with autophagy markers LC3-II, together with gene silencing and pharmacological experiments, indicate that autophagy proteins enhance viral replication and progeny virus yields. In BTV, the viral NS3 may act like the membrane protein of enveloped viruses and is responsible for intracellular trafficking and budding of virus particles [71]. Virostatics targeting virus egress would be predicted to prevent or slow down virus release and spread in an infected host, thus allowing more time for the individual’s immune system to respond and control the infection [72]. Further studies on the role of autophagy during BTV progeny virus egress/dissemination may help to understand the exact mechanism of action for C003/C052.

Our results extend our previous findings that showed cluster of lead compounds that prevent BTV induced CPE [34]. While further studies might be needed to define the exact mechanism of action for the virostatic agents C003/C052, our studies showed that these compounds may not interact with host apoptotic machinery, rather posses virostatic activity which block various signals to induce host apoptosis. The finding that host autophagy was involved in BTV life-cycle and C003/C052 might act on host autophagic machinery may open a new avenue to identify and develop novel small molecule antivirals that specifically interact with host autophagy machinery.

## Materials and Methods

### Cells, Virus Stock and Use of Compounds

BSR cells [73], a cloned derivative of baby hamster kidney (BHK) cells, were maintained in Dulbecco’s modified Eagle’s medium (DMEM) (Invitrogen, Carlsbad, CA, USA), supplemented with 5% fetal calf serum (FCS), 100 U/ml penicillin and 100 µg/ml streptomycin (Invitrogen). For all assays, unless otherwise noted, cells were plated in DMEM with optimized conditions as previously determined, including 1% FCS [34]. All cells were incubated and grown at 37°C, 5% CO_2_. The type 10 BTV (BTV-10) was plaque purified and propagated as described previously [1].

BTV-10 was titrated by standard plaque assay, stocked at 2.5×10^7^ PFU/ml and further used for infection with the indicated multiplicity of infection (MOI). For virus replication, cell monolayers were washed with FCS-free growth medium and then incubated with viruses at the indicated MOI. Virus adsorptions were carried out for 2 h at 37°C, 5% CO_2_, followed by incubation in growth medium supplemented with 1% FCS. At various hours post infection (h.p.i.), cells and supernatants were collected. Cells were sonicated for 40 s and cell debris were removed by low-speed centrifugation (2,500 g). The virus titers in the resulting supernatants were determined on BSR cells at 37°C, 5% CO_2_, using a standard plaque assay as described previously [74].

### CPE-based Cell Viability Assay

The CPE-based cell viability assays, using the CellTiter-Glo® Luminescent Cell Viability kit (Promega Inc., Madison, WI), were carried out as described previously [34]. Cell viability was determined at indicated time points using CellTiter-Glo reagents following manufacturer’s instruction (Promega). Briefly, CellTiter-Glo buffer and the lyophilized CellTiter-Glo substrate (Promega) were thawed and equilibrated to room temperature prior to use. The homogeneous CellTiter-Glo reagent solution was reconstituted after mixing the lyophilized enzyme/substrate and the buffer reagent according to the manufacturer’s instructions. Meanwhile, the assay plates were also equilibrated to room temperature for 15 min. An equal volume (25 µl) of CellTiter-Glo reagents was added to each well by a MicroFlo select dispenser (BioTek, Winooski, VT). After incubated for 15 minutes at room temperature, luminescence signals were measured using Synergy-II multi-mode microplate reader (BioTek) with an integration time of 0.1 s.

### The Ten-concentration Dose Response Assay and Cytotoxicity Assays

The virostatic efficacy assay for each compound was carried out under the optimized conditions as described previously [34]. Briefly, BSR cells were seeded into a 384-well black plate (Corning) using the MicroFlo select dispenser (BioTek). After 2 hours incubation at the 37°C, 5% CO_2_ and 80–95% humidity, ten different concentrations of each compound were serially diluted and added to each well. Cells were then infected with BTV at MOI of 0.01 *via* the Bio-Tek dispenser. Each compound treatment group, with BTV infection at denoted MOIs, includes eight replicates. Mock infection (cell only) and virus infection only controls were also included as positive and negative controls, respectively. Compound cytotoxicity assay were carried out in the same fashion, except without BTV infection. After incubated at 37°C, 5% CO_2_ and 80–95% humidity for 72 h, cell viability was determined using CellTiter-Glo reagents as described previously.

### Time-of-Addition Assay

The Time-of-Addition assay was performed to further understand the virostatic activity of the previously identified compounds [34]. Briefly, BSR cells were seeded and incubated at 37°C, 5% CO_2_ and 80–95% humidity. Cells were then inoculated with BTV at MOI of 0.01, and incubated at the optimized conditions as described previously [34]. Selected virostatic compounds were diluted to the indicated concentrations and added to each well at different time pre- or post- BTV infection. The denoted −2 and −1 h.p.i. indicate that BSR cells were incubated with the selected virostatic compounds prior to BTV infection. For 0 h.p.i., the compound and BTV were added to the culture simultaneously. Treated cells were incubated at 37°C, 5% CO_2_ with 80–95% humidity. In parallel, cells without compounds and virus treatments were included as the mock infection control. Cells with compound treatment only were served as the control to monitor compound cytotoxicity. Cells with BTV infection but without compound treatment were also included as the virus infection only control. Each BTV and compound treatment group includes eight replicates. After incubated for 72 h, cell viability was determined using CellTiter-Glo reagent as described above. The ability of virostatic compound in inhibiting BTV-induced CPE were normalized using controls designated in the assay, where cell viability in the mock infected cells was designated as 100%, and cell viability in BTV infected cells as 0%.

### The Caspase-Glo® 3/7 Apoptosis Assay

The Caspase-Glo® 3/7 apoptosis assay was accomplished using the Caspase-Glo® 3/7 assay kit (Promega) following the manufacturer’s instruction. Cell plating and incubation conditions, as well as the plate layout, were similar to that used in the CPE-based assay. BTV infection and compound addition were also in the similar fashion as described in the CPE-based virostatic efficacy assay. Caspase-Glo® 3/7 buffer and lyophilized Caspase-Glo® 3/7 substrate were equilibrated to room temperature before use. The Caspase-Glo® 3/7 buffer was transferred into the amber bottle containing Caspase-Glo® 3/7 substrate, and then mix by swirling or inverting the contents until the substrate is thoroughly dissolved to form the Caspase-Glo® 3/7 reagent. The premixed Caspase-Glo 3/7 reagents (Promega) were added at 1∶1 ratio (reagent : media) to the treated cells at 48 h.p.i., or otherwise noted in the text. After 40 minutes incubation in the dark at room temperature, luminescent signals were read with the Synergy-II multimode microplate reader (BioTek).

### Real-time Quantitative Reverse Transcriptase PCR (qRT-PCR)

Total RNA was extracted from cells and supernatants as described for TRIzol LS Reagent (Invitrogen; Carlsbad, CA) according to the manufacturer’s instructions. Dried RNA pellets were re-suspended in 50 µl DEPC treated-water and stored at −80°C before RT- PCR amplification. Following the manufactures’ instruction, the real-time qRT-PCR was carried out using the “one step SRBY Ex Taq qRT-PCR" kit (Takara, Japan). The real-time qRT-PCR was performed in a single tube and amplified products were monitored in real-time using the 7500 Fast Real-Time PCR System (AB applied biosystems). Two BTV-specific primers were designated and used for the real-time qRT-PCR:

Forward primer: 5′-GATTGATGTTTACAGGGATGAGGT-3′

Reverse primer: 5′-TCTTCCTCTGCTTGCGTCCT- 3′

### SDS-PAGE and Western Blot Analysis

Samples for SDS-PAGE and western blot analysis were prepared after boiled in protein dissociation buffer [10% (v/v) β-mercaptoethanol, 10% (wt/vol) SDS, 25% (v/v) glycerol, 10 mM Tris-HCl, pH 6.8), and 0.02% (wt/vol) bromophenol blue] for 10 min. Protein samples were loaded in 1× sample buffer and electrophoresis carried out on 12.5% SDS-polyacrylamide gels. Proteins were electroblotted on to Immobilon-P poly-vinylidene difluoride (PVDF) membrane (Millipore, Billerica, MA, USA) at 100 mA for 2 h by using a semidry electrotransfer apparatus. The PVDF membranes were immediately placed into 5% skimmed milk in TBS−0.1% Tween-20 (TBST) and incubated overnight. This step was followed by 1 h incubation in primary antibody diluted with 3% BSA in TBS. Mouse monoclonal anti-VP6 [75], mouse monoclonal anti-human LC3-II (Abgent, Inc., San Diego, CA) were used at the concentrations recommended by the manufacturers. Blots were washed three times for 10 min each in TBST and then incubated for 1 h in horseradish peroxidase-conjugated secondary antibody diluted in 3% BSA–TBST. Goat anti-mouse or anti-rabbit secondary antibodies conjugated to horseradish peroxidase (BD Biosciences, San Jose, CA) were used at 1∶5,000. Bound antibodies were detected by an enhanced chemiluminescence kit (Amersham, Piscataway, NJ, USA).

### Plaque Reduction Assay

The plaque reduction assay was carried out in the 24-well plates by including compounds in the overlay agarose, which was applied directly to the BTV infected cell culture as described previously [76], [77]. Cells were initially inoculated with BTV at ten-fold series dilution with known initial BTV titer. After absorbing for 2 h at 37°C, 5% CO_2_ with 80–95% humidity, the inoculation was removed, washed with media and replaced by 1% agarose mixed with compound at the indicated concentration, ranging from 20.0 µM to 0.16 µM. Each concentration was triplicated. At 72 h.p.i., the number of plaques formed at different compound concentration was counted and the final virus titer was determined accordingly.

### *De novo* Compound Synthesis

The structure and modification of C003 were illustrated in Figure 2 and 3. The preparation of intermediate compound **4** (IC**4**) followed an adaptation of the Gewald reaction [78] beginning with commercially available compound 4-methylacetophenone. IC**7** and IC**8** were achieved by modification of the Horner-Wadsworth-Emmons procedure [79] on commercially available 2-thiophenecarboxaldehyde and 2-pyridinecarboxaldehyde followed by a standard acid chloride formation with thionyl chloride. Combining IC**4** with IC**7** and IC**8** was pursued as described in the literature [79], [80] for related systems. Standard saponification of the resultant IC**9** and IC**10** produced IC**2b** and IC**3b** that became the final products C052 (IC**2a**) and C055 (IC**3a**) upon routine acidification (Fig. 3). All compounds were characterized by high field NMR (Bruker AM 400) and high resolution mass spectral (Waters Q Premier API Quadrupole TOF Tandem mass spectrometer) analyses with purity confirmed by micro analytical elemental determinations.
